# Evaluation of Sepsis Algorithmic Alerts Among Hospitalized Pediatric Patients in Qatar: A Retrospective Cohort Study

**DOI:** 10.1002/hsr2.72588

**Published:** 2026-09-09

**Authors:** Ghadeer Mustafa, Lorna Cynthia Lorenzo, Muna Atrash, Mary Ann Tabios, Ayat Alsmadi, Khalid Sayed Elithy, Bashir Ali Yousif, Hala Elkuni Mohamed, Tejas Grishkumar Mehta, Parwaneh Shibani, Fatma Elayouti, Kalpana Singh

**Affiliations:** ^1^ Director of Nursing Pediatrics Hamad Medical Corporation Al Wakra Qatar; ^2^ Nurse Educator Pediatric Nursing Hamad Medical Corporation Al Wakra Qatar; ^3^ Quality and Patient Safety Specialist Hamad Medical Corporation Al Wakra Qatar; ^4^ Charge Nurse Pediatric Inpatient Department Hamad Medical Corporation Al Wakra Qatar; ^5^ Senior Consultant Pediatric Intensive Care Unit, Department of Pediatrics Hamad Medical Corporation Al Wakra Qatar; ^6^ Senior Consultant Pediatric Emergency Department, Department of Pediatrics Hamad Medical Corporation Al Saad Doha Qatar; ^7^ Consultant Pediatric Inpatient, Department of Pediatrics Hamad Medical Corporation Al Wakra Qatar; ^8^ Executive Director of Nursing Hamad Medical Corporation Al Wakra Qatar; ^9^ Head Nurse Pediatric Emergency Department Hamad Medical Corporation Al Wakra Qatar; ^10^ Senior Epidemiologist Hamad Medical Corporation Doha Qatar

**Keywords:** algorithmic alert, pediatric sepsis, sensitivity and specificity, sepsis alert, sepsis bundle, sepsis protocol, SIRS

## Abstract

**Background and Aims:**

Sepsis is a leading cause of morbidity and mortality among children globally and poses a significant challenge for early detection, particularly in pediatric populations where symptoms can be subtle. An automated system for screening algorithm for sepsis is built into the electronic health record (EHR) to assist in identifying sepsis ahead of time. This study aimed to evaluate the accuracy of an algorithmic sepsis alert system for early recognition of true or suspected sepsis among pediatric patients admitted to the Pediatric Intensive Care Unit (PICU) and Pediatric Inpatient Unit at Al Wakra Hospital in Qatar.

**Materials and Methods:**

A retrospective cohort study was conducted using electronic health records of pediatric patients admitted between 01st Jan and 31st Dec 2019. Patients up to 14 years old with algorithmic alerts or clinical suspicion of sepsis were included. The study assessed the performance of algorithmic alerts, clinical suspicion, and physician judgment against defined sepsis criteria using sensitivity, specificity, predictive values, and ROC curve analysis.

**Results:**

Among 1922 patients, with an average age of 30.2 ± 37.0 months and an average hospital stay of 3.7 ± 3.3 days, 56% were male, and 85.2% were admitted to the pediatric inpatient unit. Algorithmic alerts for potential sepsis were positive in 659 patients (34.3%) upon admission, while 35 patients (1.8%) had positive clinical suspicion. Physicians judged 114 patients (5.9%) as having sepsis, and 220 (11.4%) met the criteria for true sepsis. The algorithmic alert had 9.7% sensitivity and 87.6% specificity, while clinical suspicion had 34.3% sensitivity and 89% specificity. Management as sepsis showed 21.5% sensitivity; physician judgment had 26.3% sensitivity. Without alerts, clinical suspicion and management as sepsis had 11.1% sensitivity.

**Conclusion:**

The current pediatric sepsis alert system demonstrates low sensitivity but relatively high specificity. Clinical suspicion and physician judgment showed moderate improvements in sensitivity. Adjusting the algorithmic criteria may enhance early detection and reduce delays in management. A combined approach integrating algorithmic alerts with clinical evaluation is recommended to improve sepsis identification in pediatric patients.

## Summary

**What Is Already Known**

- Sepsis is a major cause of morbidity and mortality in children worldwide and leads to significant healthcare costs.
- Early detection of sepsis is critical in pediatric care, but identifying true sepsis can be challenging due to non‐specific symptoms.

What This Study Adds

- This study evaluates an algorithmic sepsis alert system and highlights its low sensitivity but high specificity in detecting true sepsis in pediatric patients.
- It proposes potential improvements to the algorithmic criteria to enhance detection accuracy and reduce ICU admission delays for severe sepsis cases.

## 1 Introduction

Sepsis remains a leading cause of hospitalization, morbidity, and mortality among children globally. Early identification and treatment are essential for improving outcomes, yet recognizing sepsis in pediatric patients remains challenging due to age‐specific physiological responses and often subtle clinical presentations [1]. Pediatric vital signs and developmental norms vary significantly with age, making it difficult to apply standardized criteria. Unlike adults, children may initially compensate through elevated heart rates and perfusion, masking clinical deterioration until hypotension and mental status changes occur, typically in later stages of illness [2]. Diagnosing pediatric sepsis is further complicated by non‐specific symptoms and variability in disease progression. These limitations often result in delayed or missed diagnoses, as well as overtreatment in children who may not have bacterial infections [3]. As a result, recent efforts have focused on identifying reliable, early biomarkers and standardized clinical pathways to support timely recognition and response.

International guidelines, including those by the National Institute for Health and Care Excellence (NICE), advocate bundled care approaches that include early recognition tools, timely resuscitation, and ongoing performance monitoring. “*International guidelines, including those in the United Kingdom, the National Institute for Health and Care Excellence”* (nice.org.uk 2016). To assist with identifying sepsis ahead of time, many emergency departments (EDs) have started using sepsis screening tools that look for children who are more likely to get sepsis based on their vital signs, lab results, and other factors [4]. In alignment with these recommendations, Hamad Medical Corporation (HMC) in Qatar implemented a pediatric sepsis care pathway integrating system‐generated alerts based on the Systemic Inflammatory Response Syndrome (SIRS) and the Qatar Early Warning Score (QEWS).

Despite a national sepsis initiative launched in 2017, Qatar lacks published data on the accuracy of pediatric algorithmic sepsis alerts. Local observations suggest that many alerts do not correlate with actual clinical diagnoses, indicating a need to assess the system's performance. Furthermore, data from Gulf states on pediatric sepsis recognition tools remains limited.

This retrospective cohort study aims to evaluate the diagnostic accuracy of the pediatric algorithmic sepsis alert system at Al Wakra Hospital. This research study is to evaluate the accuracy of the algorithmic sepsis alert in early recognition of sepsis and/or suspected sepsis among hospitalized pediatric patients admitted to the Pediatric Intensive Care Unit (PICU) and the Pediatric Inpatient Unit of Al Wakra Hospital.

## 2 Study Design and Setting

This study employed a retrospective observational cohort design based on chart reviews of pediatric patients’ electronic health records (EHRs) at Al Wakra Hospital (AWH), a tertiary hospital under Hamad Medical Corporation (HMC) in Qatar. The evaluation focused on the diagnostic accuracy (sensitivity and specificity) of a sepsis algorithmic alert system among pediatric patients who presented to the Pediatric Emergency Department and were subsequently admitted to the Pediatric Inpatient Unit or Pediatric Intensive Care Unit (PICU). Additional data from related HMC facilities, including the Pediatric Emergency Center and Hamad General Hospital, were also considered for eligibility.

### 2.1 Study Population

The study included all pediatric patients (aged ≤ 14 years) admitted to AWH's Pediatric Inpatient Unit or PICU between January 1 and December 31, 2019. Patients were eligible if they triggered a sepsis alert or were suspected of having sepsis during hospitalization.

### 2.2 Inclusion and Exclusion Criteria

Patients aged 14 or younger admitted to the PICU or Pediatric Inpatient Unit at AWH during the study period were included. We excluded admissions to pediatric units other than PICU or inpatient wards, as well as cases located only in the NICU, due to the unique nature of neonatal sepsis.

### 2.3 Sampling and Sample Size

A census sampling approach was adopted. All eligible patient encounters meeting the inclusion criteria during the 12‐month study period were reviewed. The final sample included 1922 pediatric admissions: 1499 from the inpatient ward and 423 from the PICU.

Since this study involved a retrospective chart review of the entire eligible population over a specified period, no prior sample size calculation or power analysis was performed. The study has approximately 88–90% power to detect a difference between the observed sensitivity (9.7%) and a clinically meaningful alternative sensitivity of 20%, at a significance level of 0.05, using 220 confirmed sepsis cases.

### 2.4 Data Collection

**Figure 1:**
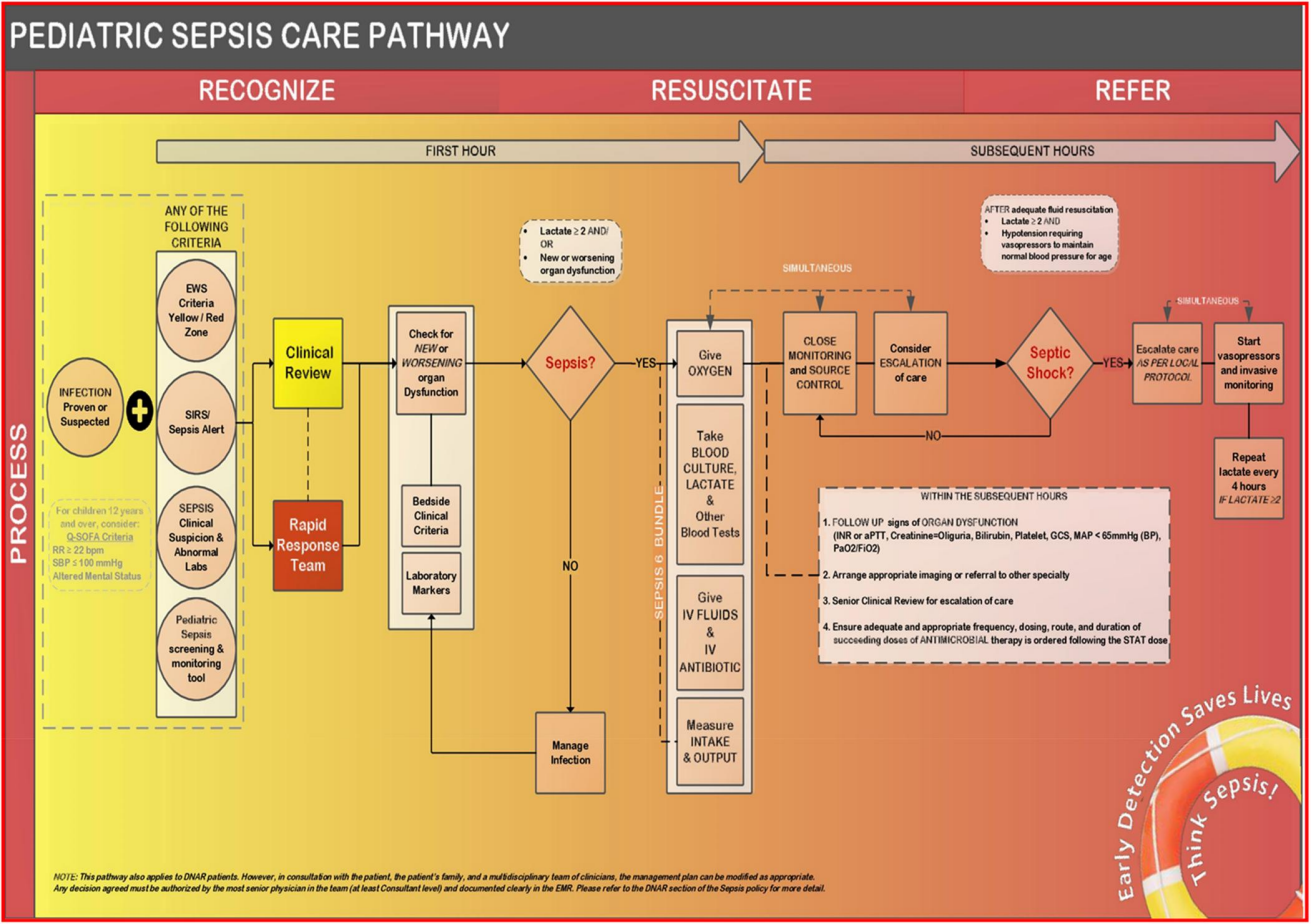
Pediatric Sepsis Care Pathway Tool taken from 2018 to 2022 CPW 10314.

**Figure 2:**
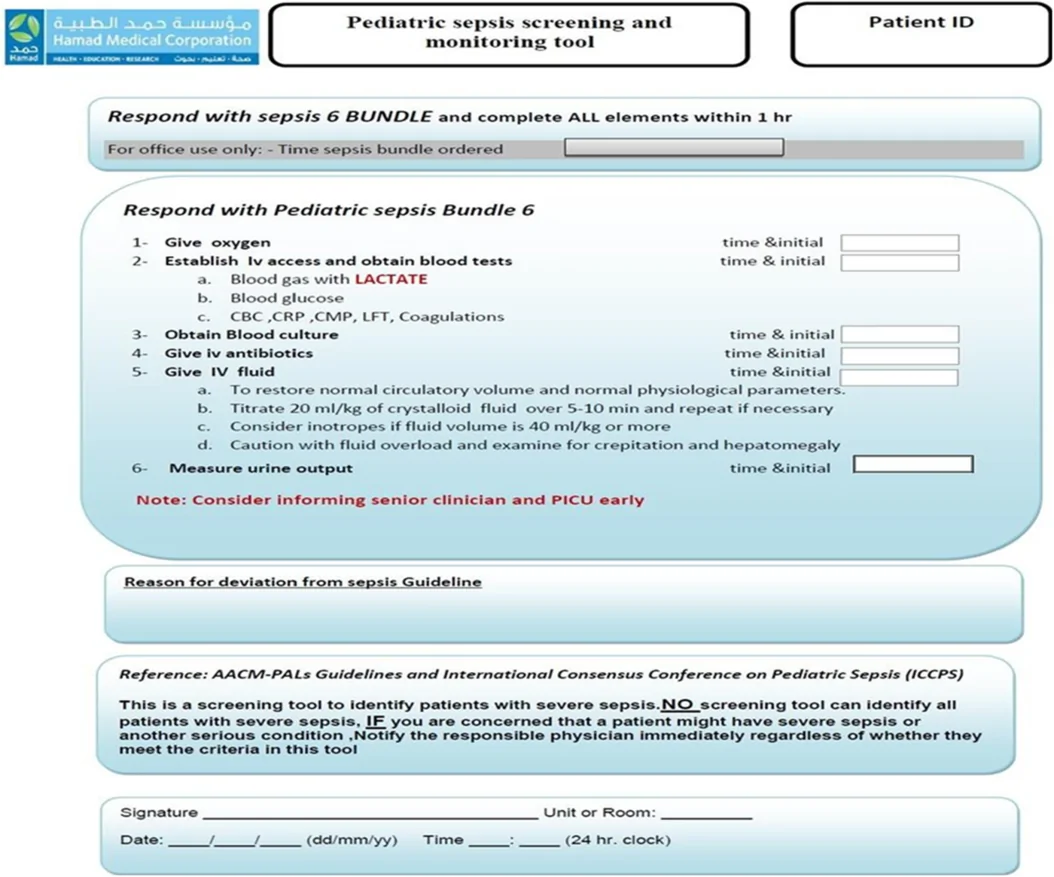
Pediatric Screening Monitoring tool taken from 2018 to 2022 CPW 10314.

A retrospective review of electronic health records (EHR) was conducted for all patients admitted from January 1st, 2019, to December 31st, 2019, to evaluate suspected or confirmed sepsis. Al Wakra Hospital uses the Cerner EHR system, incorporating the pediatric sepsis care pathway criteria based on the St. John Sepsis Surveillance Agent [5]. This system, developed by Cerner in 2010, uses vital parameters from the Pediatric Early Warning Score and laboratory values from Harriet Lane to identify and stratify sepsis patients. Figure 1 shows the sepsis care pathway, in which a pathway is created to guide the healthcare providers on the immediate action in patients with suspected or confirmed sepsis. Figure 2 displays the Pediatric Screening Monitoring Tool aligned with the 6 bundles in ongoing performance monitoring from the [6] guidelines.

Patient was stratified into two groups: first group with sepsis algorithmic alert, second group with clinical suspicion of sepsis, either by nurse, as proven by creation of sepsis notification form or clinical suspicion by physician using sepsis order set or equivalent orders. The physician assesses the patient for sepsis according to the hospital's clinical practice pathway. A spreadsheet was developed to extract targeted parameters from the Cerner database. These data were reviewed by a physician for sepsis onset in pediatric inpatients and by a pediatric clinician for those managed as suspected or confirmed sepsis in the PICU.

The information collected included demographic data, vital signs, mental status, perfusion, algorithmic sepsis alert, and activation of the sepsis order set or its equivalent, which involves microbiology culture and antibiotic administration. The cultures ordered included blood, cerebrospinal fluid, urine, pus, or wound cultures, along with at least one intravenous or oral antibiotic. Additional data included lactate levels, intravenous fluids, oxygen administration, urine output, and cellular elements like white blood cell count, platelet count, creatinine, bilirubin, and glucose, with higher values indicating severe organ dysfunction. Equivalent order sets must include microbiology culture and antibiotics to be reviewed.

### 2.5 Pediatric Sepsis Algorithmic Alerts

The clinical workflow includes the following process: (1) Immediate algorithmic sepsis alert if the patient's vital signs and biomarkers hit the built‐in SIRS/Sepsis criteria. Thresholds for SIRS will trigger if 3 or more of the following five criteria will be satisfied: (1) temperature < 36 or > 38.5 degree Celsius; (2) heart rate < 90 or > 180 beats/min; (3) respiratory rate < 25 or > 50 breath/min; (4) white blood cells < 5 or > 17.5 nanoliter; or (5) glucose > 7.8 and < 11.1 mmol/L. Threshold for severe sepsis was established when 2 or more SIRS criteria present, and 1 or more of the following four organ system dysfunction will be satisfied; (1) cardiovascular system: Systolic blood pressure of < 65 mmHg and/or MAP < 55 mmHg; (2) tissue perfusion: serum lactate > 2.0 mmol/L; (3) hepatic system: total bilirubin: > 34.2 and < 171 mmol/L; and (4) renal system: serum creatinine: > 44.2 mmol/L. Alert notifications with clinical indications are sent to the assigned nurse, who informs the physician.

Physician judgment is used when a doctor reviews all patient medical records in retrospect to ensure that they fit all requirements. They then complete the Electronic Health Record (EHR) sepsis notification form, selecting “Sepsis Ruled Out Yes” if the criteria for sepsis or suspected sepsis are not met, or “Sepsis Ruled Out No” if sepsis or suspected sepsis is confirmed. If indicated, the physician orders the Golden Hour set, initiating the sepsis care bundle within 60 min. Upon receiving a notification from nursing staff that an alert had “fired,” clinical suspicion of sepsis can be raised by a physician using the sepsis order set or equivalent orders without a prior alert, or by a nurse generating a sepsis notification sent to the assigned physician without a system alert.

**Figure 3:**
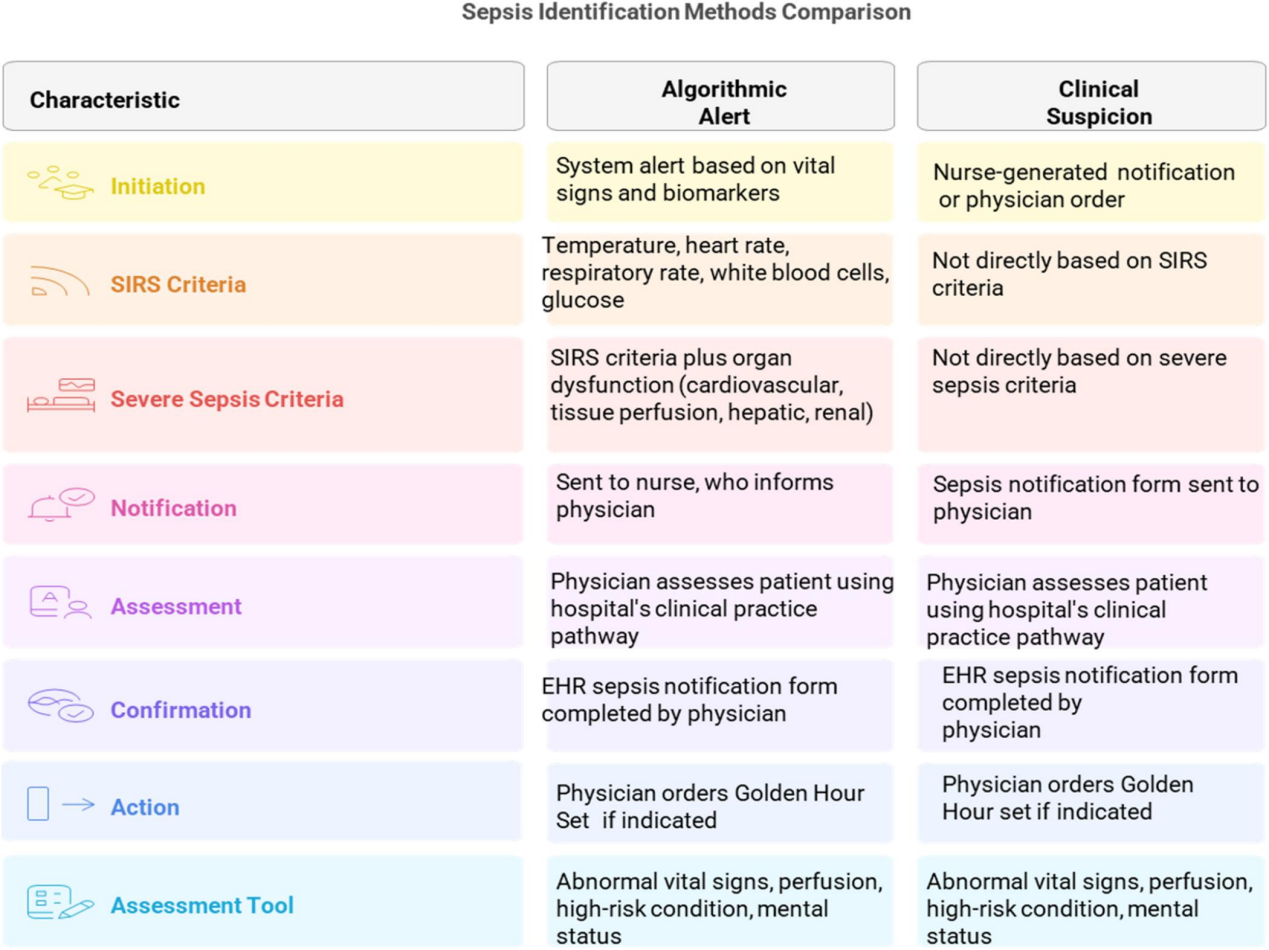
Sepsis alert algorithm.

In order to help their clinical diagnosis, pediatricians typically relay the clinical picture of sepsis and a positive blood, CSF or other specific culture. The physician then conducts a sepsis assessment using the hospital's clinical practice pathway screening tool. This involves checking for abnormalities in any two vital signs plus at least one of the following: abnormal perfusion (capillary refill > 2 s), high‐risk condition, and mental status (lethargic, unresponsive, non‐responsive to stimuli, agitated) (Figure 3).

Any additional orders will be considered part of the equivalent set and reviewed for sepsis confirmation. The first 24 h post‐activation of the order set or equivalent will be examined for care escalation and Rapid Response Team (RRT) activation. Discharge diagnoses will be reviewed for sepsis‐related codes per ICD‐10. Severe sepsis or suspected sepsis is determined using all available clinical and laboratory information in the EHR by the physician. Strong correlations were found between a number of indicators of organ dysfunction and outcomes. In children with infections, lower levels of consciousness and higher Pediatric Risk of Mortality (PRISM) scores were significantly linked to the onset of sepsis or severe sepsis, indicating that early neurological decline and increased severity scores are crucial indicators of disease advancement. Examples of correlation of organ dysfunctions and outcomes are lower level of consciousness and higher Pediatric Risk of Mortality scores in children who have an infection are linked to sepsis or severe sepsis; in children who have sepsis or severe sepsis/septic shock, chronic conditions, oncologic diagnosis, use of vasoactive/inotropic agents, mechanical ventilation, serum lactate, platelet count, fibrinogen, procalcitonin, multi‐organ dysfunction syndrome, Pediatric Logistic Organ Dysfunction score, Pediatric Index of Mortality‐3, and Pediatric Risk of Mortality score all showed significant and reliable relationships with mortality which shows a true positive definition of sepsis that person has the disease and the test is positive [7, 8]. Overall, these findings show that signs of organ dysfunction and validated pediatric severity scoring systems have strong predictive relationships with poor outcomes. This supports their use in finding true positive cases of pediatric sepsis, where the disease is present, and the diagnostic indicators are also positive.

### 2.6 Statistical Analysis

The study evaluated the performance of three identification methods, including physician judgment, an algorithmic alert and an algorithm alert plus manages as sepsis. In the analysis of continuous variables, such as vital signs, the median with IQR, mean, and SD were used to summarize the data. For categorical variables, proportions were used to present the data. To compare the performance of the identification methods, the area under the receiver operating characteristic (ROC) curve was calculated. Performance metrics were calculated for each identification method, including sensitivity, specificity, positive and negative predictive values, and positive and negative likelihood ratios. These metrics provide information on the accuracy and reliability of each method in correctly identifying visits for patients with sepsis/septic shock. The 95% confidence interval (CI) was also calculated to provide an estimate of the precision of these metrics. All data were analyzed using STATA 17. software (StataCorp. 2017).

## 3 Results

### 3.1 Participants Characteristics

A total of 1922 pediatric patients were included in this retrospective study. The median age was 16.0 months (IQR: 3.0, 48.0), and the median length of stay was 3.0 days (IQR: 2.0, 4.0). Among the participants, 56% were male, and 85.2% were admitted to the pediatric inpatient unit. Demographic and clinical baseline characteristics are presented in Table 1.

**Table 1:** Participant Characteristics.

Variables	Level	Value
*N*		1922
Age in months, median (IQR)		16.0 (3.0, 48.0)
Gender	F	846 (44.0%)
	M	1076 (56.0%)
Admission Unit		
Pediatric Inpatient Unit		1639 (85.2%)
Pediatric ICU		285 (14.8%)
Length of stay Median (IQR)		3.0 (2.0, 4.0)

Out of 1922 patients evaluated, 34.3% (*n* = 659) had a sepsis alert triggered at the time of admission, while 65.7% (*n* = 1263) did not. Among all patients, only 1.8% (*n* = 35) had no alert but were still clinically suspected of sepsis, indicating that the alert system captured the majority of suspected sepsis cases Table 2.

**Table 2:** Sepsis alert at the admission.

Variables	Total
*N*	1922
Sepsis alert at the time of admission	659 (34.3%)
No alert, Clinical Suspicion of Sepsis	35 (1.8%)

### 3.2 Sepsis Alerts and Clinical Suspicion

Of the 1922 patients, 659 (34.3%) triggered a positive algorithmic alert for potential sepsis upon admission, while only 35 patients (1.8%) were identified through clinical suspicion alone. Vital signs recorded at admission included temperature, heart rate, respiratory rate, oxygen saturation, blood pressure, mental status, and capillary refill (Table 3). Laboratory and microbiological investigations included cultures (blood, CSF, urine, wound), serum lactate, white blood cell count, platelets, creatinine, bilirubin, and glucose levels, all considered indicative of infection severity and possible organ dysfunction. The sepsis alert system was linked to initiation of the “Sepsis 6” bundle, including IV antibiotics, fluids, oxygen, and diagnostic testing.

**Table 3:** Triggering criteria for sepsis vital signs.

	Sepsis alert at the time of admission	No alert, clinical suspicion of sepsis	Managed as sepsis without clinical suspicion and sepsis alert
*N*	659	35	18
Temperature	171 (26.1%)	15 (42.9%)	9 (50%)
Temperature, mean (SD)	37.6 (0.9)	37.8 (1.3)	38.1 (0.9)
Heart rate	182 (27.9%)	16 (45.7%)	10 (56%)
Heart rate, mean (SD)	129.2 (23.5)	148.1 (20.4)	154.7 (28.4)
Respiratory rate	187 (28.6%)	12 (34.3%)	5 (28%)
Respiratory rate, mean (SD)	36.4 (9.9)	36.4 (9.9)	
Oxygen Saturation	14 (2.1%)	1 (2.9%)	1 (6%)
Blood pressure	50 (8.2%)	4 (13.3%)	1 (7%)
Systolic, mean (SD)	96.1 (8.7)	93.8 (13.4)	96.5 (8.6)
MAP, mean (SD)	70.9 (6.9)	73.2 (11.5)	—
Mental status	8 (1.2%)	2 (5.7%)	—
Capillary refill	4 (1.5%)	1 (2.9%)	—

Label: Vital signs documented that triggered the pediatric sepsis/SIRS alert.

### 3.3 Utilization of Sepsis Order Set Components Across Different Patient Groups

Among the 659 patients with a sepsis alert at admission, 80.3% had blood cultures collected, 91.2% had urine cultures, and only 2.4% had CSF cultures. In contrast, 91.4% of those with no alert but with clinical suspicion of sepsis had blood cultures, and 54.3% had urine cultures; notably, 25.7% had CSF cultures, a higher proportion than the alert group. In the group managed as sepsis without alert or clinical suspicion (*n* = 18), all received blood cultures, 50% had urine cultures, and 50% had CSF cultures. Lactate testing was performed in only 19.7% of the alert group but in 77.1% of the clinically suspected group and 50% of the unflagged managed group. IV fluids were administered to 32.8% of patients with an alert, but to 91.4% of the clinically suspected group and 72% of the managed group. Oxygen therapy was used in 29.9% of the alert group, 65.7% of the clinically suspected group, and 39% of the managed group. Notably, documentation of urine output was low in the alert group (only 35.5% recorded as “Yes” and 55.1% as undocumented), while it was better captured in the other groups. Only 8.2% of alert patients were formally managed as sepsis using either the sepsis order set or an equivalent, compared to 100% in the other two groups (Table 4).

**Table 4:** Characteristic of sepsis order set.

Variables	Sepsis alert at the time of admission	No alert, clinical suspicion of sepsis	Managed as sepsis without clinical suspicion and sepsis alert
*N*	659	35	18
Blood Culture	529 (80.3%)	32 (91.4%)	18 (100%)
Urine Culture	601 (91.2%)	19 (54.3%)	9 (50%)
CSF Culture	16 (2.4%)	9 (25.7%)	9 (50%)
Wound Culture	108 (16.4%)	2 (5.7%)	0 (0.0%)
Lactate	130 (19.7%)	27 (77.1%)	9 (50%)
IV Fluid	216 (32.8%)	32 (91.4%)	13 (72%)
Oxygen	197 (29.9%)	23 (65.7%)	7 (39%)
Urine Output	234 (35.5%)	25 (71.4%)	10 (56%)
Equivalent Order set	13 (1.9%)	0 (0.0%)	18 (100%)
Managed as Sepsis (Order set & Equivalent Order set)	54 (8.2%)	35 (100.0%)	18 (100%)

Label: Interventions that were done or not done based on the sepsis orders set.

### 3.4 Sepsis Management and Diagnosis

Among all patients, 107 (5.6%) were managed as sepsis based on both alert and clinical suspicion, and 18 (0.94%) were treated as sepsis despite the absence of both. A total of 114 patients (5.9%) were confirmed to have true sepsis based on physician judgment. A review of order sets and clinical actions within the first 24 h of alert activation or equivalent orders assessed escalation of care and Rapid Response Team (RRT) involvement (Table 5).

**Table 5:** Criteria for True Sepsis starting in positive culture result.

Variables	Label: 6 bundles	Sepsis alert at the time of admission	No alert, clinical suspicion of sepsis	Managed as sepsis without clinical suspicion and sepsis alert
*N*		659	35	18
Blood		35 (6.4%)	0 (0.0%)	2 (11.1%)
Urine		69 (22.4%)	3 (11.1%)	3 (30.0%)
CSF		1 (0.5%)	0 (0.0%)	1 (25.0%)
Pus/Wound		9 (56%)	0 (0%)	0 (0.0%)
Positive Culture Result (1,2,3 combination of any c/s)	0	557 (84.5%)	32 (91.4%)	14 (77.8%)
	1	92 (14.0%)	3 (8.6%)	2 (11.1%)
	2	8 (1.2%)	0 (0.0%)	2 (11.1%)
	3	2 (0.3%)	0 (0.0%)	0 (0.0%)
Upgrade Antibiotic		60 (9.1%)	2 (5.7%)	1 (5.6%)
WBC		345 (53.1%)	7 (21.2%)	2 (11.1%)
WBC, mean (SD)		15.1 (11.2)	12.8 (7.3)	10.8 (6.7)
Platelet		135 (21.2%)	4 (12.1%)	4 (22.2%)
Platelet, mean (SD)		364.5 (154.2)	387.0 (128.4)	343.6 (207.5)
Creatinine		68 (10.5%)	9 (27.3%)	3 (16.7%)
Creatinine, mean (SD)		30.0 (16.0)	37.8 (24.5)	33.1 (13.6)
Bilirubin		34 (5.9%)	12 (38.7%)	2 (12.5%)
Bilirubin, mean (SD)		14.5 (33.1)	64.7 (90.2)	16.4 (27.6)
Glucose		125 (19.3%)	1 (3.0%)	5 (27.8%)
Glucose, mean (SD)		6.6 (2.8)	5.4 (1.6)	8.0 (6.7)
Lactate		100 (41.8%)	19 (63.3%)	4 (44.4%)
Lactate, mean (SD)		2.2 (1.7)	3.2 (3.1)	2.3 (1.6)
Transfer/Escalation Within 24 hrs to a higher level of care		26 (3.9%)	9 (25.7%)	7 (38.9%)
RRT/Code activation		40 (6.1%)	6 (17.1%)	7 (38.9%)
No. of criteria met as True sepsis (Laboratory Result)	Zero criteria	123 (18.7%)	5 (14.3%)	6 (33.3%)
	One criterion	210 (31.9%)	7 (20.0%)	1 (5.6%)
	Two criteria	200 (30.3%)	11 (31.4%)	6 (33.3%)
	Three criteria	81 (12.3%)	8 (22.9%)	0 (0.0%)
	Four criteria	36 (5.5%)	2 (5.7%)	2 (11.1%)
	Five criteria	6 (0.9%)	1 (2.9%)	0 (0.0%)
	Six criteria	2 (0.3%)	1 (2.9%)	2 (11.1%)
	Seven criteria	1 (0.2%)	0 (0.0%)	0 (0.0%)
	Eight criteria	0 (0.0%)	0 (0.0%)	1 (5.6%)
True Sepsis (Physician's Judgment)		60 (9.1%)	8 (22.9%)	4 (22.2%)

### 3.5 Diagnostic Accuracy of Identification Methods

The diagnostic performance of various identification methods was assessed using sensitivity, specificity, and predictive values. The algorithmic alert showed a sensitivity of 9.7% (95% CI: 7.6–12.2) and specificity of 87.6% (95% CI: 85.7–89.4). Clinical suspicion achieved higher sensitivity at 34.3% (95% CI: 19.1–52.2) with specificity of 89.0% (95% CI: 87.5–90.4). Cases managed as sepsis had a sensitivity of 21.5% (95% CI: 14.1–30.5) and specificity of 89.1% (95% CI: 87.6–90.5), while physician‐confirmed sepsis demonstrated sensitivity of 26.3% (95% CI: 18.5–35.4) and specificity of 89.5% (95% CI: 88.0–90.9). In patients managed as sepsis without alert or suspicion, sensitivity dropped to 11.1% (95% CI: 1.4–34.7) and specificity was 88.3% (95% CI: 86.3–90.0). Full diagnostic metrics, including likelihood ratios and predictive values, are detailed in Tables 6 and 7.

**Table 6:** Two‐by‐two tables that demonstrate performance of sepsis alert and clinical suspicion vs managed as sepsis.

Sepsis alert vs criteria			Clinical suspicion vs criteria		
	Criteria +	Criteria −		Criteria +	Criteria −
Alert +	64	595	clinical suspicion+	12	23
Alert −	156	1107	clinical suspicion −	208	1679

Managed as sepsis vs criteria			Physician judgment vs criteria			Without managed as sepsis, no alert and no clinical suspicion vs criteria		
	Criteria +	Criteria −		Criteria +	Criteria −		Criteria +	criteria −
Alert +	23	84	Physician judgment +	30	84	No alert & no clinical suspicion +	2	16
Alert −	197	1618	Physician judgment −	190	1617	No alert & no clinical suspicion −	142	1068

**Table 7:** Test characteristics of sepsis screening test.

Test	Test characteristics with 95% CI					
	Sepsis alert vs criteria		Clinical suspicion vs criteria	Managed as sepsis vs criteria	Physician judgment vs criteria	Without alert, clinical suspicion and managed as sepsis vs criteria
Sensitivity (%)	9.7 (7.6 to 12.2)		34.3 (19.1 to 52.2)	21.5 (14.1 to 30.5)	26.3 (18.5 to 35.4)	11.1 (1.4 to 34.7)
Specificity (%)	87.6 (85.7 to 89.4)		89 (87.5 to 90.4)	89.1 (87.6 to 90.5)	89.5 (88 to 90.9)	88.3 (86.3 to 90)
Positive Predictive value (%)	29.1 (23.2 to 35.6)		5.5 (3.9 to 9.3)	10.5 (6.7 to 15.3)	13.6 (9.4to 18.9)	1.4 (0.17 to 4.9)
Negative predictive value (%)	65 (62.7 to 67.3)		98.6 (98 to 99.1)	95.1 (93.9 to 96.0)	95.1 (93.9 to 96)	98.5 (97.6 to 99.2)
Positive likelihood ratio	0.78 (0.60 to 1.04)		3.1 (1.9 to 5.01)	1.98 (1.35 to 2.91)	2.5 (1.8 to 3.5)	0.95 (0.25 to 3.53)
Negative likelihood ratio	1.03 (1.00 to 1.06)		0.74 (0.58 to 0.94)	0.88 (0.80 to 0.97)	0.82 (0.74 to 0.92)	1.01 (0.85 to 1.19)
Receiver operative characteristic curve data	0.49 (0.47 to 0.50)		0.62 (0.54 to 0.70)	0.55 (0.51 to 0.59)	0.58 (0.54 to 0.62)	0.50 (0.42 to 0.57)

### 3.6 Receiver Operating Characteristic (ROC) Analysis

**Figure 4:**
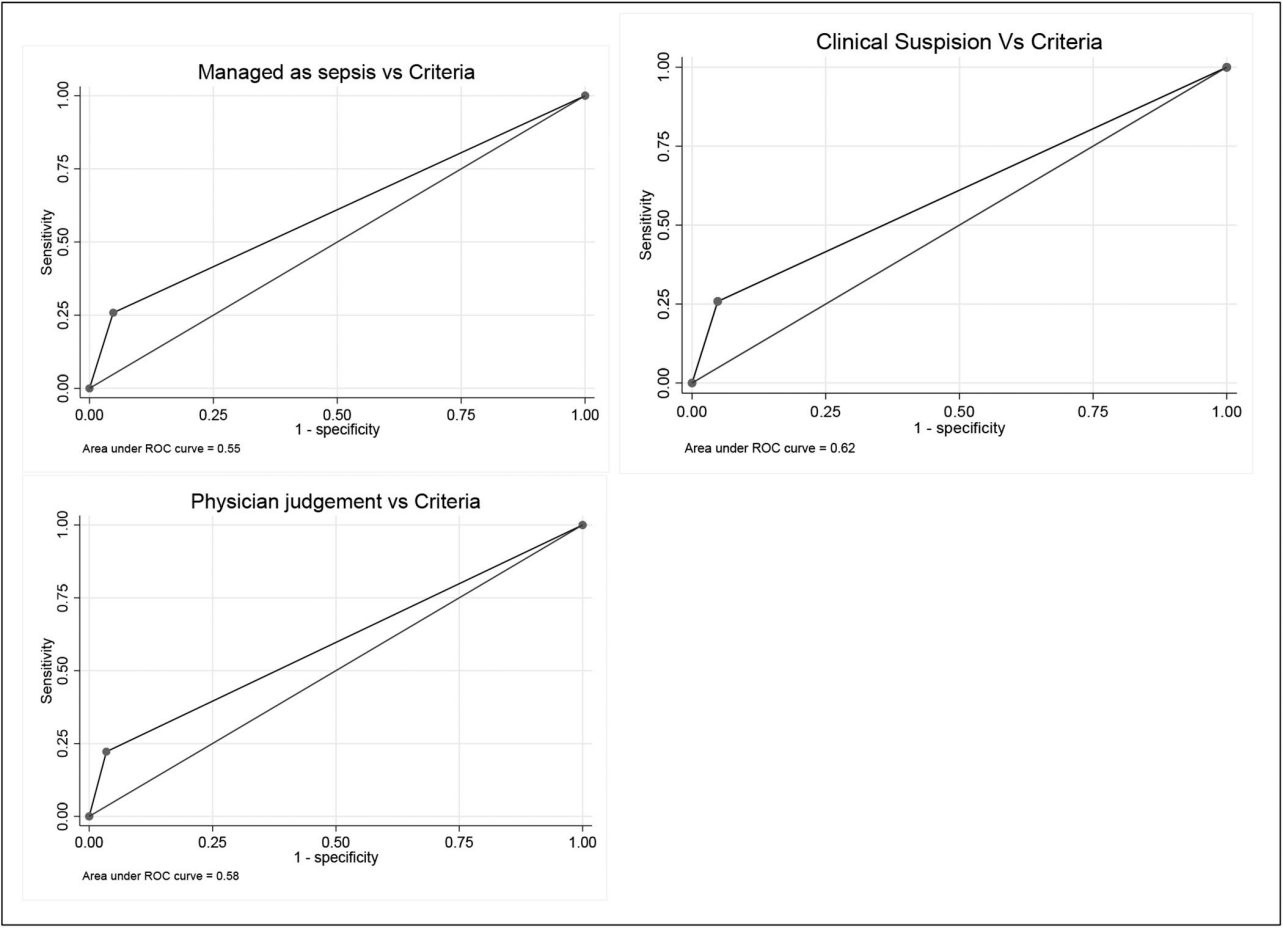
Area under the curve for sepsis screening.

The Area Under the Receiver Operating Characteristic Curve (AUROC) was calculated for each identification approach to assess overall discriminatory ability. The algorithmic alert yielded an AUROC of 0.49 (95% CI: 0.47–0.50), indicating poor performance. Clinical suspicion showed better discrimination with an AUROC of 0.62 (95% CI: 0.54–0.70). The AUROC for managed as sepsis was 0.55 (95% CI: 0.51–0.59), and for physician judgment, it was 0.58 (95% CI: 0.54–0.62). The approach involving no alert or suspicion but managed as sepsis yielded an AUROC of 0.50 (95% CI: 0.42–0.57), indicating no better performance than chance Figure 4.

## 4 Discussion

This study evaluated the accuracy and clinical relevance of pediatric sepsis detection strategies—specifically, algorithmic alerts, clinical suspicion, and physician judgment at Al Wakra Hospital. Our findings show considerable limitations in the current system's ability to identify true sepsis cases in children, raising concerns about both under‐ and over‐detection.

A key observation was the high frequency of algorithmic alerts (34.3%) compared to the low proportion of patients identified through clinical suspicion (1.8%) or ultimately confirmed with sepsis (5.9%). This discrepancy suggests that the algorithm may produce excessive false positives, consistent with previous research on alert fatigue in sepsis detection systems [9, 10]. Alert fatigue—where clinicians become desensitized to frequent, low‐value alerts—can hinder timely response to actual sepsis cases and reduce trust in automated systems [10, 11].

Although clinical suspicion showed higher sensitivity than algorithmic alerts, it still failed to detect many true cases of sepsis. This highlights the limitations of relying solely on clinical judgment, especially in pediatric patients who often present with non‐specific symptoms [12]. Our findings reinforce the need for a hybrid approach: combining the broad screening capacity of algorithms with the contextual interpretation provided by clinicians [13, 14]. Vital signs and laboratory results including lactate levels, white blood cell count, creatinine, and glucose—were recorded to assess organ dysfunction and inform management decisions. These parameters have been consistently associated with early sepsis detection in pediatric populations [15, 16, 17]. Despite the collection of this data, the study revealed that only a small fraction (5.6%) were managed as sepsis cases with both alert and suspicion present. An even smaller group (0.94%) was treated as sepsis without any system alert or clinical concern, raising further questions about consistency in recognition and treatment protocols.

In terms of diagnostic accuracy, the sepsis alert system showed low sensitivity (9.7%) and an AUROC of 0.49, indicating it performed no better than random chance. Clinical suspicion had better sensitivity (34.3%) and an AUROC of 0.62, while physician judgment and management decisions fell in between. These findings echo prior studies that emphasized the limitations of both machine‐driven alerts and unassisted clinical judgment when used in isolation [18, 19]. Overall, this study underscores the importance of integrating multiple modalities to improve pediatric sepsis detection. While alerts can serve as useful prompts, they require refinement to reduce false positives. Clinical judgment remains essential but insufficient when used alone. Future research should aim to develop enhanced, context‐aware algorithms that incorporate real‐time vitals, lab data, and individualized risk profiles to improve early and accurate identification.

This study has several limitations that must be considered when interpreting the findings. First, the study was conducted at a single tertiary facility, Al Wakra Hospital in Qatar, which may limit generalizability to other healthcare settings with different patient demographics, clinical workflows, or sepsis management protocols. Multi‐center studies across diverse populations and institutions would help validate and expand upon these results. Second, the study focused exclusively on the Cerner St. John Sepsis Agent. While this system is widely used, it does not reflect the performance of other sepsis detection algorithms, such as the Epic Sepsis Model or machine learning‐driven tools. A comparative evaluation of multiple alert systems would provide a broader perspective on diagnostic utility. Third, as a retrospective observational study, we were constrained by the quality and completeness of existing electronic health record data. Unmeasured confounders, missing clinical context, or provider decision‐making factors may have influenced both alert performance and clinical actions. Finally, we were unable to perform multivariable adjustment or subgroup stratification due to data limitations, which may affect the precision of sensitivity and specificity estimates. Despite these constraints, the study provides critical insights into the real‐world performance of pediatric sepsis alert systems and identifies key areas for improvement and further research.

## 5 Conclusion and Recommendation

This study highlights the challenges of detecting pediatric sepsis using standalone tools such as algorithmic alerts or clinical suspicion. Although the alert system demonstrated high specificity, it suffered from poor sensitivity and low predictive accuracy, limiting its utility as a standalone diagnostic method. Our findings suggest that combining alerts with physician oversight improves identification of true sepsis cases, but further refinement of the algorithm is needed to enhance sensitivity without compromising specificity.

We recommend a systematic revision of algorithmic thresholds and integration of machine learning approaches that can dynamically adapt to pediatric patient profiles. Implementing a hybrid detection model that leverages both technological tools and clinical expertise may lead to earlier and more accurate sepsis recognition, ultimately improving patient outcomes. Future prospective studies are needed to validate revised models and assess their impact on care quality and mortality reduction.

## Author Contributions

**Ghadeer Mustafa:** conceptualization, methodology, supervision, writing – original draft, writing – review and editing. **Lorna Cynthia Lorenzo:** data curation, writing – original draft, writing – review and editing. **Muna Atrash:** data curation, writing – original draft, conceptualization, methodology, supervision. **Mary Ann Tabios:** data curation, writing – original draft. **Ayat Alsmadi:** data curation, writing – original draft. **Khalid Sayed Elithy:** supervision, validation, methodology, writing – original draft. **Bashir Ali Yousif:** conceptualization, validation, project administration, resources, writing – original draft. **Hala Elkuni Mohamed:** project administration, methodology, writing – original draft. **Tejas Grishkumar Mehta:** supervision, methodology, validation, writing – original draft. **Parwaneh Shibani:** project administration, writing – original draft, resources, investigation, validation. **Fatma Elayouti:** writing – original draft, data curation, investigation. **Kalpana Singh:** methodology, software, validation, formal analysis, supervision, writing – original draft, visualization, writing – review and editing.

## Ethics Statement

Ethical considerations were considered, and the study adhered to the guidelines and principles outlined in the “Declaration of Helsinki.” Approval was obtained from the IRB of Hamad Medical Research Center (MRC) with the number MRC‐01‐21‐781. No Informed consent was obtained from subjects as it is retrospective study.

## Conflicts of Interest

The authors declare no conflicts of interest.

## Transparency Statement

The lead author Ghadeer Mustafa affirms that this manuscript is an honest, accurate, and transparent account of the study being reported; that no important aspects of the study have been omitted; and that any discrepancies from the study as planned (and, if relevant, registered) have been explained.

## Data Availability

The authors confirm that the data supporting the findings of this study are available within the article. Additional data is available from the corresponding author upon reasonable request. Due to ethical and institutional restrictions, the raw data is not publicly available, as they contain sensitive patient information collected from electronic health records. Access to the data is subject to approval by the Hamad Medical Corporation Institutional Review Board.
